# New Heterotrinuclear Cu^II^Ln^III^Cu^II^ (Ln = Ho, Er) Compounds with the Schiff Base: Syntheses, Structural Characterization, Thermal and Magnetic Properties

**DOI:** 10.3390/ma15124299

**Published:** 2022-06-17

**Authors:** Beata Cristóvão, Dariusz Osypiuk, Agata Bartyzel

**Affiliations:** Department of General and Coordination Chemistry and Crystallography, Institute of Chemical Sciences, Faculty of Chemistry, Maria Curie-Sklodowska University in Lublin, Maria Curie-Sklodowska Sq. 2, 20-031 Lublin, Poland; dariusz.osypiuk@mail.umcs.pl (D.O.); agata.bartyzel@mail.umcs.pl (A.B.)

**Keywords:** compartmental Schiff base, heteronuclear Cu^II^Ln^III^Cu^II^ complex, thermal analysis TG/DSC, TG-FTIR, ATR-FTIR spectroscopy

## Abstract

New heterotrinuclear complexes with the general formula [Cu_2_Ln(H_2_L)(HL)(NO_3_)_2_]·MeOH (Ln = Ho (**1**), Er (**2**), **H_4_L** = *N*,*N*′-bis(2,3-dihydroxybenzylidene)-1,3-diaminopropane) were synthesized using compartmental Schiff base ligand in conjugation with auxiliary ligands. The compounds were characterized by elemental analysis, ATR-FTIR spectroscopy, X-ray diffraction, TG, DSC, TG-FTIR and XRD analysis. The N_2_O_4_ salen-type ligand coordinates 3*d* and 4*f* metal centers via azomethine nitrogen and phenoxo oxygen atoms, respectively, to form heteropolynuclear complexes having CuO_2_Ln cores. In the crystals **1** and **2**, two terminal Cu(II) ions are penta-coordinated with a distorted square-pyramidal geometry and a Ln^III^ ion with trigonal dodecahedral geometry is coordinated by eight oxygen atoms from [Cu^II^(H_2_L)(NO_3_)]^−^ and [Cu^II^(HL)(NO_3_)]^2−^ units. Compounds **1** and **2** are stable at room temperature. During heating, they decompose in a similar way. In the first decomposition step, they lose solvent molecules. The exothermic decomposition of ligands is connected with emission large amounts of gaseous products e.g., water, nitric oxides, carbon dioxide, carbon monoxide. The final solid products of decomposition **1** and **2** in air are mixtures of CuO and Ho_2_O_3_/Er_2_O_3_. The measurements of magnetic susceptibilities and field dependent magnetization indicate the ferromagnetic interaction between Cu^II^ and Ho^III^ ions **1**.

## 1. Introduction

**Figure 1:**
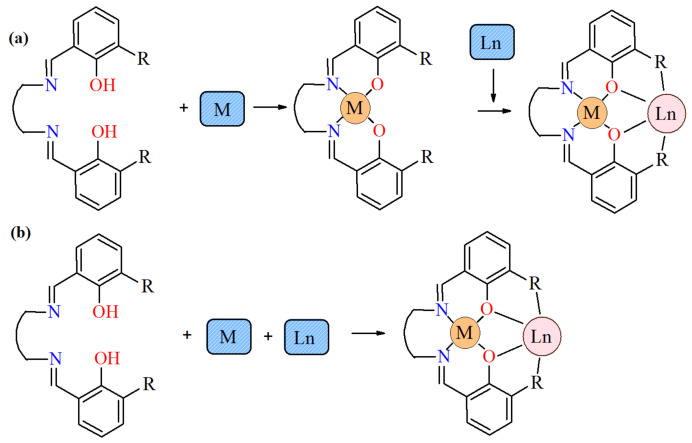
Methods synthesis of salen-type Schiff base heteronuclear complex: (**a**) a stepwise reaction; (**b**) one-pot reaction (where R = substituent with O-donor atom).

In recent years, much progress in the synthesis and investigation of heteronuclear 3*d*–4*f* Schiff base coordination compounds has been observed [1,2,3,4,5,6,7,8,9,10,11,12,13,14,15,16,17,18]. Their crystal structures and properties are determined by several factors, e.g., type of the metal ions, metal-to-ligand stoichiometry, the nature and position of the ligands, methods of synthesis (a stepwise reaction or one-pot reaction, Figure 1), type of solvents, kind of coligands, etc.

**Figure 2:**
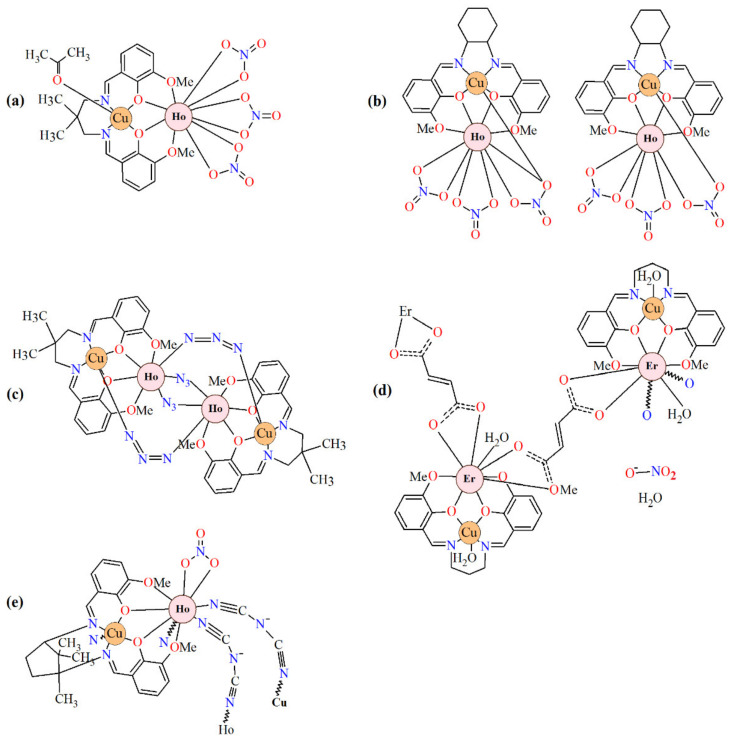
Chemical diagrams of selected heteronuclear complexes Cu^II^-Ho^III^/Er^III^ including salen–type Schiff base ligands: (**a**) [Cu(L)(ace)Ln(NO_3_)_3_], (**b**) [Cu(L)Ln(NO_3_)_3_], (**c**) [Cu_2_(valdmpn)_2_Ln_2_(N_3_)_6_]·2(MeOH)_0.5_, (**d**) [LCu^II^Er^III^(H_2_O)_2_(fum)]NO_3_·3H_2_O, (**e**) [LCuHo(dca)_2_(NO_3_)]_n_ (where Ln = Ho, Er).

The salen-type Schiff bases are ligands obtained from salicylaldehyde or its derivatives and different diamines. They usually consist of rigid aromatic rings and flexible aliphatic chains [1,2,3,4,5,6,7,8,9,10,11,12,13,14,15,16,17,18,19]. In the coordination compounds formed by them, 3*d* and 4*f* metal ions are captured simultaneously and linked together through two O_phenol_ atoms. Lanthanide(III) ions behave as hard acids and prefer oxygen to nitrogen donors, whereas 3*d* metal ions may coordinate to both nitrogens and oxygens. The following salen-type Schiff bases: *N*,*N*′-bis(3-methoxysalicylidene)-1,3-diamino-2,2-dimethylpropane [5], *N*,*N*′-bis(3-methoxysalicylidene)cyclohexane-1,2-diamine [8], *N*,*N*′-bis(3-methoxysalicylidene)-1,3-diamino-2,2-dimethylpropane [11], *N*,*N*′-propylenedi(3-methoxysalicylideneimie) [14], 1*R*,3*S*)-*N*′,*N*″-bis[3-methoxysalicylidene]-1,3-diamino-1,2,2-trimethylcyclopentane [17], that differ by kind of diamines, were used in the synthesis of heteronuclear Cu^II^Ho^III^/Er^III^ complexes. The reported compounds (Figure 2) prepared in different ways and conditions are characterized by interesting structures and properties [5,8,11,14,17].

Among them, there are stepwise synthesized heterobinuclear complexes [Cu(L)(ace)Ln(NO_3_)_3_] (where Ln = Ho^III^, Er^III^, ace = acetone, H_2_L = *N*,*N*′-bis(3-methoxysalicylidene)-1,3-diamino-2,2-dimethylpropane) (Figure 2a). In the crystals Cu^II^ and Ho^III^/Er^III^, ions are doubly bridged with the phenolate oxygen atoms. The magnetic investigations of the compounds indicated the presence of the ferromagnetic coupling between the Ho^III^/Er^III^ and Cu^II^ spins [5]. The chiral *N*,*N*′-bis(3-methoxysalicylidene)cyclohexane-1,2-diamine (H_2_L) was applied for the synthesis of the dinuclear complexes [Cu(L)Ln(NO_3_)_3_] (where Ln = Ho^III^, Er^III^). The isomorphous complexes are composed of two diphenoxo-bridged Cu^II^Ln^III^ dinuclear clusters (Figure 2b). In the molecular structure, each Cu^II^ center adopts a distorted square pyramid geometry. In opposite to them, the two Ln^III^ ions have different coordination environments: one is ten- the other is nine-coordinated. Magnetic investigations of Cu^II^Ln^III^ indicate that Cu^II^Ho^III^ exhibits field-induced slow magnetic relaxation behaviors [8]. The azido-bridged copper(II)–lanthanide(III) heterotetrametallic complexes [Cu_2_(valdmpn)_2_Ln_2_(N_3_)_6_]·2(MeOH)_0.5_ (where Ln = Ho, Er, H_2_valdmpn = *N*,*N*′-bis(3-methoxysalicylidene)-1,3-diamino-2,2-dimethylpropane) were prepared during a reaction of a metalloligand Cu(valdmpn), a respective lanthanide(III) chloride and sodium azide in methanol. Their structures contain isolated tetranuclear [CuLLn]_2_ clusters where the Ho^III^/Er^III^ centers are bridged by two end-on (EO) azides (Figure 2c). Magnetic investigations of Cu^II^_2_Ho^III^_2_/Er^III^_2_ revealed the SMM behavior for the Cu^II^_2_Ho^III^_2_ complex [11].

The heterometallic coordination polymer _∞_[LCu^II^Er^III^(H_2_O)_2_(fum)]NO_3_·3H_2_O was obtained during reaction of [LCuEr(NO_3_)_3_] (L = *N*,*N*′-propylenedi(3-methoxysalicylideneiminato) and fumaric acid (H_2_fum). In the crystals, the {CuEr} nodes (Figure 2d) are connected by fum^2−^ bridges (coordinated only to the lanthanide(III) ions). The magnetic studies show that the values χ_M_T decrease on lowering the temperature [14]. The compound [LCuHo(dca)_2_(NO_3_)]_n_ (L = double deprotonated (1*R*,3*S*)-*N*′,*N*″-bis[3-methoxysalicylidene]-1,3-diamino-1,2,2-trimethylcyclopentane, dca = dicyanamide) is composed of a dinuclear [LCuHo]^3+^ moiety, one nitrate anion and two bridging dca. Similarly to compounds described above in its crystal structure, the Cu^2+^ and *4f* ions are linked by two *μ*-phenoxo oxygen atoms of the Schiff base ligand (Figure 2e). The complex is a potential molecule-based multifunctional material indicating optical, ferromagnetic and ferroelectric properties [17]. Literature reported magnetic properties of heteronuclear 3*d*–4*f* complexes shows the influence of the lanthanide type on the magnetic exchange coupling interactions between Ln^III^ and paramagnetic 3*d* metal ions. The mechanism of the 3*d*–4*f* interaction are the subject of many studies [20,21,22,23,24,25,26,27]. According to the theoretical model suggested by Kahn et al., the coupling should be antiferromagnetic for the Ln(III) of the first half of the lanthanide row (*n* < 7) and ferromagnetic for the Ln(III) of the second half of the lanthanide row (*n* ≥ 7). In determining the magnetic properties of 3*d*–4*f* complexes, the orbital angular momentum and spin orbit coupling of unpaired 4*f*-electron plays a crucial role. In the case of Ln^III^ with 4*f*^1−6^ configuration, angular and spin moments are antiparallel in the ^2S + 1^*L_J_* free-ion ground state (*J = L−S*). A parallel alignment of the Cu^II^ and Ln^III^ spin moment would result in an antiparallel alignment of the angular moment, that is, to an overall antiferromagnetic interaction. For Ln^III^ with configuration 4*f*^7−13^ (*J = L + S*), a parallel alignment of the Cu^II^ and Ln^III^ spin moment would lead to an overall ferromagnetic interaction [20]. The investigations of magneto-structural correlation indicate that the exchange interaction in 3*d*–4*f* compounds is governed by the value of the dihedral angle between OMO and OLnO planes. For higher value of this angle, the weaker coupling between 3*d* and 4*f* metal ions should be anticipated. Superexchange contribution can be awaited for coordination compounds with a planar LnO_2_M molecular fragment [21,22,23,24,25,26,27].

As a continuation of the investigation on salen-type Schiff base complexes, the aim of this work was to obtain heteronuclear species with *N*,*N*′-bis(2,3-dihydroxybenzylidene)-1,3-diaminopropane (the ligand is characterized by the presence in a *meta* position of a benzene ring –OH substituent instead of –OCH_3_) and study their properties, as well as investigate the influence of the kind of the additional functional groups in the ligand and kind of lanthanide(III) ions on the structure and feature of the 3*d*–4*f* compounds. So far, starting from the *N*,*N*′-bis(2,3-dihydroxybenzylidene)-1,3-diaminopropane and respective Cu^2+^ and Ln^3+^ salts, we have synthesized heteropolynuclear complexes with different structures and physicochemical properties [28,29,30]. In the case of the first half of the lanthanide row (La^III^, Pr^III^, Nd^III^), the inert heterotrinuclear compounds Cu^II^Ln^III^Cu^II^ which differ only in the amount and type of solvent molecules in the outside coordination sphere were obtained. In the crystals of copper(II) and praseodymium(III)/neodymium(III) complexes, the antiferromagnetic coupling of magnetic centers occurred [28]. The hexanuclear cation complex resulted from the simultaneous coordination of two dianionic Schiff bases to Cu^II^ and Gd^III^ ions and the forming of trinuclear units [Cu^II^_2_Gd^III^] that were connected through bridging nitrate ions. The interaction between neighboring Cu^II^ and Gd^III^ ions was ferromagnetic [29]. We also obtained and characterized the heterodinuclear Cu^II^Dy^III^ compound. Its magnetic measurements showed the weak ferromagnetic interaction between Cu^II^ and Dy^III^ ions [30]. It was noticed that in the heterodi-, heterotri- and heterohexanuclear complex crystals reported by us so far, the *N*,*N*′-bis(2,3-dihydroxybenzylidene)-1,3-diaminopropane was double deprotonated.

Herein, we report the synthesis and crystal characterization, along with the spectral, thermal and magnetic properties of new heterotrinuclear compounds [Cu_2_Ln(H_2_L)(HL)(NO_3_)_2_]·MeOH (where Ln = Ho, Er) contained in the crystals dianionic H_2_L^2−^ and trianionic HL^3−^ Schiff base ligands. The complexes were synthesized in a step-wise manner without the isolation of the mononuclear complex.

## 2. Materials and Methods

### 2.1. Materials

The reagent grade chemicals, i.e., 2,3-dihydroxybenzaldehyde (HO)_2_C_6_H_3_CHO, 1,3-diaminopropane NH_2_(CH_2_)_3_NH_2_, Ho(NO_3_)_3_·5H_2_O, Er(NO_3_)_3_·5H_2_O, Cu(CH_3_COO)_2_·H_2_O were used.

### 2.2. Synthesis

#### 2.2.1. *N*,*N*’-Bis(2,3-dihydroxybenzylidene)-1,3-diaminopropane (**H_4_L**)

The ligand **H_4_L** was prepared from 1,3-diaminopropane (5 mmol, 0.37 g) and 2,3-dihydroxybenzaldehyde (10 mmol, 1.38 g) in 50 mL of hot methanol according to literature procedures [31]. Yield 80%. *Anal*. (%) C_17_H_18_N_2_O_4_. Calcd: C, 64.97; H, 5.73; N, 8.92%. Found: C, 65.20; H, 5.70; N, 9.10%.

#### 2.2.2. Complexes [Cu_2_Ln(H_2_L)(HL)(NO_3_)_2_]·MeOH (**1**, **2**)

The complexes were prepared following the same general procedure: a methanol solution (10 mL) of copper acetate monohydrate (0.4 mmol, 0.0799 g) was added to a hot methanol solution (50 mL) of the Schiff base **H_4_L** (0.4 mmol, 0.1248 g) to produce a green colored mixture which was magnetically stirred. After 30 min, a methanol solution (5 mL) containing dissolving Ho(NO_3_)_3_·5H_2_O (0.2 mmol, 0.0887 g) or Er(NO_3_)_3_·5H_2_O (0.2 mmol, 0.0882 g) was added and the resulting mixture was stirred for about 30 min. The resulting clear, deep green solutions were left undisturbed in a refrigerator at ~4 °C. The X-ray quality green crystals of the desired compounds were obtained over a period of several days.

##### Complex [Cu_2_Ho(H_2_L)(HL)(NO_3_)_2_]·MeOH (**1**)

Yield 70%, 150 mg. *Anal*. (%) for C_35_H_35_Cu_2_HoN_6_O_15_ (MW: 1071.70). Calcd: C, 39.23; H, 3.29; N, 7.84; Cu, 11.86; Ho, 15.39. Found: C, 39.40; H, 3.10; N, 7.50; Cu, 11.60; Ho, 15.50.

##### Complex [Cu_2_Er(H_2_L)(HL)(NO_3_)_2_]·MeOH (**2**)

Yield 65%, 140 mg. *Anal*. (%) for C_35_H_35_Cu_2_ErN_6_O_15_ (MW: 1074.03). Calcd: C, 39.14; H, 3.28; N, 7.82; Cu, 11.83; Er, 15.57. Found: C, 39.30; H, 3.40; N, 7.60; Cu, 11.50; Er, 15.80.

### 2.3. Methods

A CHN 2400 Perkin Elmer analyzer was used for determination of C, H and N contents. The metal amounts were determined on an ED XRF spectrophotometer (Canberra-Packard, Schwadorf, Austria). The ATR-FTIR spectra were recorded on a Nicolet 6700 spectrophotometer equipped with the Smart iTR attachment (diamond crystal) over 4000–525 cm^−1^. Thermal analysis was carried out by the thermogravimetric (TG) and differential scanning calorimetry (DSC) methods using the SETSYS 16/18 analyzer (Setaram, Lyon, France). The samples 7.61 mg (**1**) and 6.66 mg (**2**) were heated in open Al_2_O_3_ crucibles in air at the range of 20–1000 °C at a heating rate of 10 °C·min^−1^. TGA Q5000 analyzer (TA Instruments, New Castle, DE, USA) interfaced to the Nicolet 6700 FTIR spectrophotometer (Thermo Scientific, Waltham, MA, USA) were applied for the TG-FTIR analysis. The samples in an open platinum crucible were heated from room temperature to 700 °C (heating rate was 20 °C·min^−1^). The temperature in the gas cell and transfer line was set to 250 and 240 °C, respectively. XRD analysis of the solid residue was carried out by using PAN Analytical/Empyrean spectrophotometer. The dc magnetic susceptibilities of the compounds were measured on Quantum Design SQUID-VSM magnetometer in a range of 1.8–300 K. The magnetization curves were recorded at 2K in an applied field up to 5 T. Diamagnetic corrections were estimated from Pascal’s constants [32].

#### X-ray Crystal Structure Determination

Single-crystal data for the complexes were collected on an Oxford Diffraction Xcalibur CCD diffractometer (MoKα radiation, λ = 0.71073Å). The program CrysAlis [33] was used for collecting frames of data, cell refinement and data reduction. A multi-scan absorption correction was applied. Crystal data, data collection and structure refinement details are summarized in Table 1. The structures were solved by direct methods using SHELXS-2018 and refined by the full-matrix least-squares on F^2^ using the SHELXL-2018 [34] (both programs implemented in WinGX software [35]). All the non-hydrogen atoms were refined with anisotropic displacement parameters. The H-atoms attached to carbon were positioned geometrically and refined applying the riding model [C–H = 0.93–0.99 Å and with U_iso_(H) = 1.2 or 1.5 Ueq(C)]. The O-bound H atoms were located on a difference Fourier map and refined freely or with O–H distances restrained to 0.82 Å using DFIX command. The following programs were used to prepare the molecular graphics: ORTEP3 [35] and Mercury [36]. The geometrical calculations were performed using PLATON program [37].

## 3. Results and Discussion

**Figure 3:**
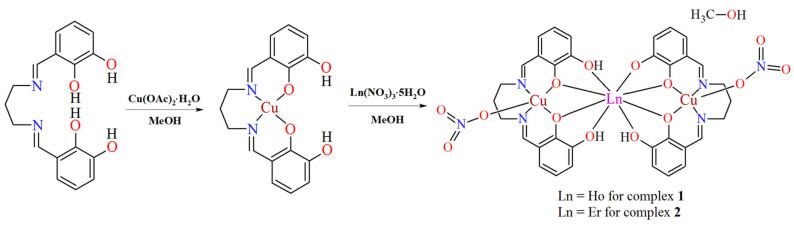
The scheme of the synthetic route of complexes **1** and **2**.

*N*,*N*′-bis(2,3-dihydroxybenzylidene)-1,3-diaminopropane **H_4_L** is a multidentate ligand which possess six donor atoms, i.e., two imino nitrogen atoms and four oxygen atoms coming from hydroxyl groups. The ligand can act as a bridge between metal ions through phenoxy groups so as to link the 3*d* and 4*f* ions together, therefore, it can be used to synthesize 3*d*–4*f* complexes. The inner, smaller N_2_O_2_ compartment of the Schiff base may accommodate a borderline acid, e.g., copper(II) ion, whereas the other, bigger O_2_O_2_ site selectively binds to hard acids, such as lanthanide(III) ion. Using **H_4_L**, the copper(II) acetate and the holmium(III)/erbium(III) nitrate, we obtained the discrete heterotrinuclear complexes of the general formula [Cu_2_Ln(H_2_L)(HL)(NO_3_)_2_]·MeOH (Ln = Ho **1**, Er **2**) (Figure 3). The neutral complexes are isostructural, crystalize with one CH_3_OH solvent molecule and are characterized by the molar ratio between the Schiff base ligand and the 3*d* and 4*f* metal ions 2:2:1. The similar values of ionic radious of Ho^III^ and Er^III^ cations and the same molar ratio of the starting compounds may be the origin of the same crystal structure of **1** and **2**.

### 3.1. Infrared Spectra

The FTIR spectra of **1** and **2** (Table 2, Figure S1) are similar. A broad absorption bands in the 2500–3300 cm^−1^ region can be attributed to the O–H stretching vibrations of methanol molecule (it interferes with the protonated hydroxyl groups of the N_2_O_4_ ligand) that are involved in the strong hydrogen bonds.

A broad absorption bands in the 2500–3300 cm^−1^ region can be attributed to the O–H stretching vibrations of methanol molecule (it interferes with the protonated hydroxyl groups of the N_2_O_4_ ligand) that are involved in the strong hydrogen bonds. This feature is in accordance with the X-ray structures, i.e., methanol molecule acts as a proton acceptor as well as a proton donor. The FTIR spectra of complexes have in common the occurrence of a strong absorption band at 1618 cm^−1^ **1** and 1616 cm^−1^ **2** which is characteristic of the presence of the azomethine group C=N. These bands are shifted towards lower frequencies relative to the free Schiff base 1632 cm^−1^. This phenomenon is due to the coordination of azomethine nitrogen to the 3*d* metal ion. The strong bands situated at 1467 cm^−1^, 1285 cm^−1^ and 1024 cm^−1^, respectively, in the spectrum of **1** and 1465 cm^−1^, 1287 cm^−1^ and 1024 cm^−1^ in the spectrum of **2** may be assigned to the monodentate nitrate ligand. The involvement of the phenolic oxygen atoms in the metal-ligand bonding is confirmed by the strong doublet bands observed at 1251 cm^−1^, 1218 cm^−1^ **1** and 1248 cm^−1^, 1219 cm^−1^ **2**, respectively. The typical absorption band of the *ν*_aryl_-O vibration is identified in the free ligand spectrum at 1233 cm^−1^ [4,13,38,39,40,41,42,43,44]. All these spectroscopic features are confirmed by the X-ray structures.

### 3.2. Crystal and Molecular Structure

**Figure 4:**
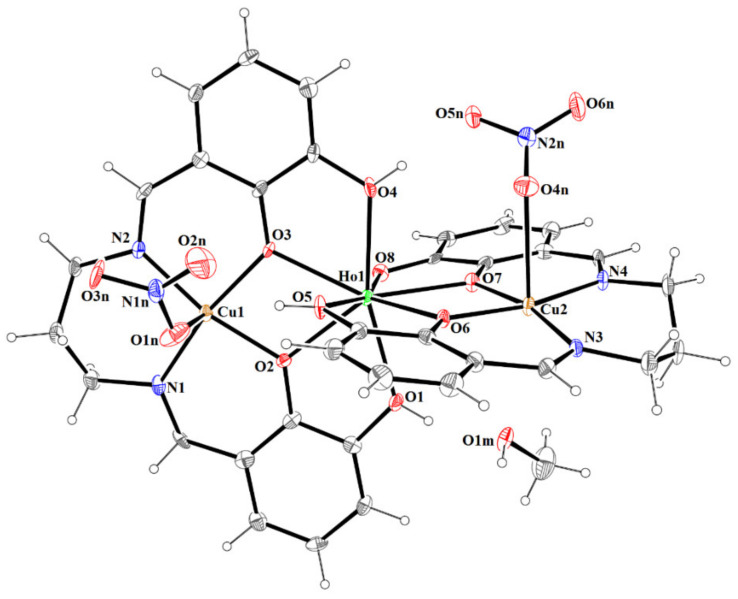
The molecular structure of **1**. Displacement ellipsoids are drawn at the 30% probability level.

The reaction of the Schiff base ligand **H_4_L** with copper(II) acetate and lanthanide(III) nitrate result in formation of the trinuclear complexes **1** and **2** which are isomorphous and crystallize in the centrosymmetric monoclinic space group *P*2_1_/*c* (Table 1). The asymmetric unit cell of both complexes contains one neutral complex, which consists of two Cu(II) ions, one Ln(III) ion, a dianionic ligand H_2_L^2−^, a trianionic ligand HL^3−^, two nitrite ions and methanol molecule (Figure 4 and Figure S2).

The complex structures are constructed from almost linear trinuclear [Cu_2_-Ln] units. The values of the Cu-Ln-Cu angle are 167.97(2)° and 168.04(2)°, respectively, for **1** and **2** (Table 3). The distances between the copper(II) and lanthanides(III) ions are within the range 3.4831(7)–3.4977(8) Å.

**Figure 5:**
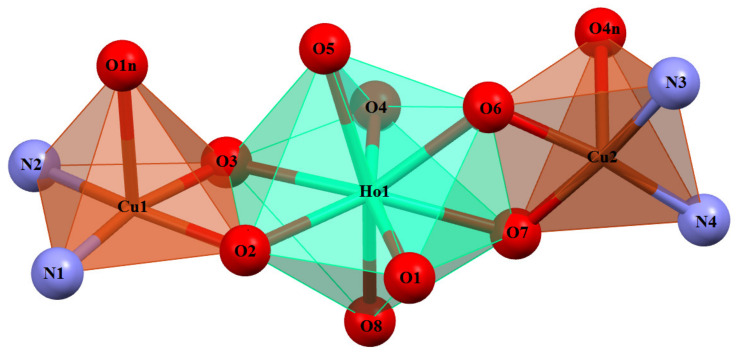
Coordination polyhedra of Cu(II) and Ho(III) cations in the trinuclear complex **1**.

The Ho^III^ and Er^III^ ion assume a trigonal dodecahedron [O_8_] configuration (Figure 5 and Figure S3), while both the partially deprotonated Schiff base ions (H_2_L^2−^ and HL^3−^) act in similar chelating coordination modes, i.e., lanthanide(III) ion is coordinated by four oxygen atoms of phenoxide or phenol groups of each ligand. A similar binding type of lanthanide (with partially deprotonated Schiff bases, i.e., one dianionic and one trianionic ligand) was reported for a trinuclear complex of Zn^II^-Tb^III^-Zn^II^ ions [45]. The Cu(1) and Cu(2) centers have slightly distorted square pyramidal coordination geometries (Figure 5 and Figure S3) in which the equatorial sites are occupied by two nitrogen and two oxygen atoms of Schiff base ligands. The Cu-O and Cu-N bonds are within normal values (C-O 1.913(4)-1.983(5) Å and C-N 1.960(5)-1.977(5) Å) and comparable to those observed in the related Cu^II^ compounds [40,43,46,47,48,49]. The apical positions are occupied by oxygen atoms from monodentate nitrate ions and the bond lengths are found to be significantly longer (distances in the range 2.410(6)-2.649(5) Å) than those of Cu-O in the basal plane.

Moreover, the Cu(2)-O bonds in the axial position are a bit longer from average distances for this kind of connection (2.45 Å) but in literature [40], there are some structures where the Cu-O_nitrate_ bond has similar or higher value. Examples of structures with refcodes and bond lengths are given in Table 4. The planes formed by N_2_O_2_ cores around Cu(II) ions of two Schiff bases intersect at an angle of 78,25(1)° for **1** and 78,20(3)° for **2**.

**Figure 6:**
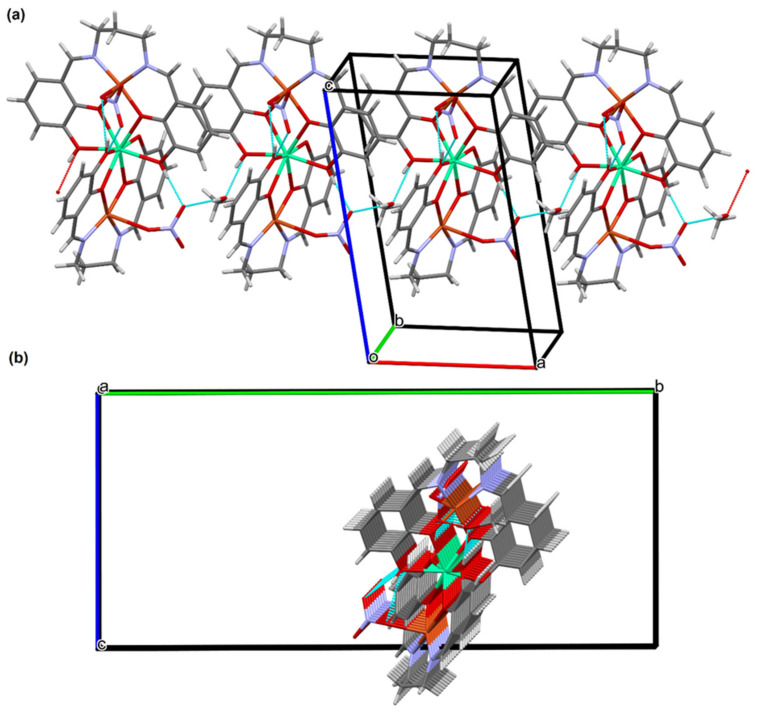
(**a**) A partial viewed along the *b*-axis direction of the crystal packing of **1** with hydrogen bonds shown as dashed lines; (**b**) A partial viewed along the *a*-axis direction of the crystal packing of **1** with hydrogen bonds shown as dashed lines.

The crystal structures of **1** and **2** reveal the presence of intramolecular and intermolecular hydrogen bonds (Table S1). In the crystals **1** and **2**, the molecules are linked by O(1)–H(1o)···O(1m) and O(1m)–H(1m)···O(5n)^a^ (symmetry code (a): x−1,y,z) hydrogen bonds forming columns propagating along [100] with $C_{2}^{2}\left(10\right)$ motifs (Figure 6 and Figure S4). Additional classical hydrogen bonds are supported by weaker non-classical C–H···O contacts, which linked formed columns in 3D supramolecular structure. The partial view of crystal packing for compound **1** and **2** are illustrated in Figures S5 and S6.

### 3.3. Thermal Analysis

**Figure 7:**
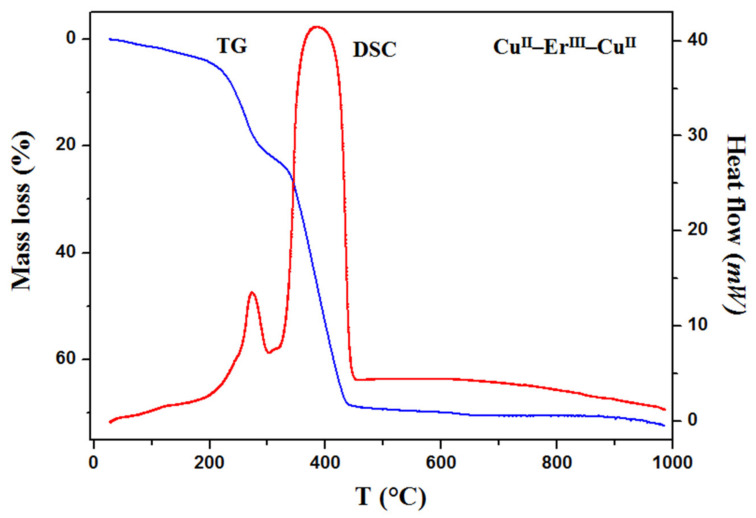
TG and DCS curves of thermal decomposition of the complex **2** in air.

In order to examine the thermal behavior of the heteronuclear complexes **1** and **2**, the thermogravimetric analysis was carried out (Figure 7 and Figure S7). The results of the thermal analysis allow to confirm/evaluate the presence of solvents (e.g., methanol, water) in the structure of compounds and to establish the endothermic and/or exothermic effects connected with different processes such as dehydration, desolvation, melting or decomposition. The TG and DSC curves recorded for both complexes are similar. The mass of samples decreases slowly with the increasing temperature. The first mass loss occurs up to 90 °C and it is assigned to the elimination of one methanol molecule (mass loss: observed 2.60% **1**, 2.70% **2**, calculated 2.99% **1**; 2.98% **2**). The small endothermic effect seen on the DSC curves confirms this process. In the case of compound **1**, the decomposition process begins immediately after desolvation. The next mass losses recorded at above 200 °C and accompanied with exothermic effects seen on the DSC curves is connected with gradual decomposition of the samples. Additionally, this process is also confirmed by the TG-FTIR analysis (Figure S8).

The recorded TG-FTIR spectra show that carbon dioxide, carbon monoxide and nitric oxide are mainly emitted during this process. The characteristic doublet bands seen at 2240–2400 cm^−1^ and 670 cm^−1^, respectively, are assigned to stretching and deformation vibrations of carbon dioxide molecules. The specific bands at 2060–2240 cm^−1^ are characteristic of carbon monoxide [59]. The solid intermediate products for thermal decomposition could not be identified. The residual mass is about 29.5% **1** and 30.6% **2** (the theoretical values are 31.5% **1** and 32.6% **2**). At high temperature, the sublimation of the copper(II) oxide takes place and the differences between values calculated and found can be caused by this process. Mixtures of metal oxides CuO and Ho_2_O_3_/Er_2_O_3_, experimentally verified by X-ray diffraction powder patterns (Figures S9 and S10) are the final solid products of thermal decomposition of **1** and **2** in air [60].

### 3.4. Magnetic Properties

**Figure 8:**
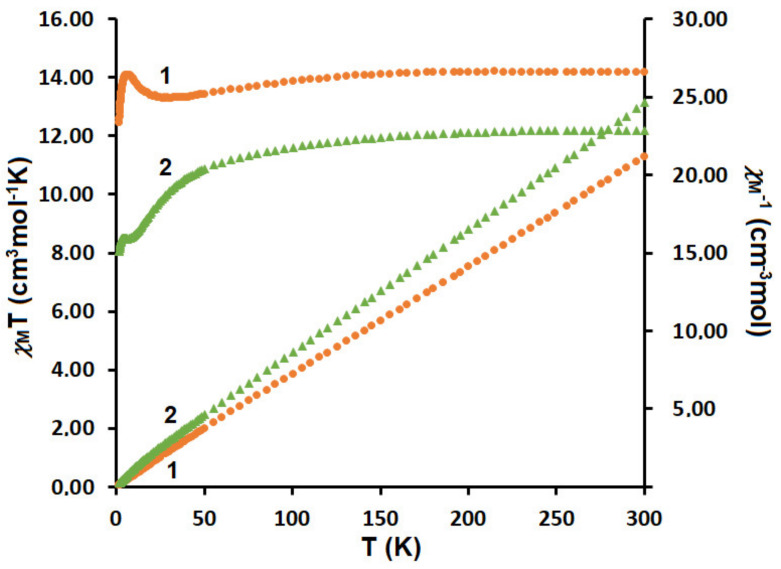
Temperature dependence of experimental χ_M_*T* and χ_M_^−1^ versus *T* for **1** and **2**.

Temperature-dependent molar susceptibility measurements of Cu^II^_2_Ho^III^ **1** and Cu^II^_2_Er^III^ **2** were carried out in a magnetic field of 0.1 T at 1.8–300 K. The χ_M_*T* vs. *T* curves for **1** and **2** are shown in Figure 8. The magnetic properties of heteronuclear Cu^II^Ln^III^ compounds are governed by three factors: the thermal population of the Stark components of Ln^III^, the Cu^II^···Cu^II^ interactions (including intermolecular interaction) and the Cu^II^Ln^III^ interactions. For Cu^II^_2_Ho^III^ **1**, the χ_M_*T* value experimentally determined at 300 K is equal to 14.17 cm^3^Kmol^−1^, which is slightly smaller than the value 14.82 cm^3^Kmol^−1^ calculated for one Ho^III^ (^5^*I*_8_, *J =* 8, *L* = 6, *S* = 2, *g* = 5/4) and the two Cu^II^ (*S* = ^1^/_2_, *g* = 2) free ions. As the temperature is lowered, χ_M_*T* keeps a constant value until 150 K, then begins to decrease to 13.91 cm^3^Kmol^−1^ at 19 K, next increases to reach a value of 14.70 cm^3^Kmol^−1^ at 5.9 K and finally, shows a small decrease to 12.95 cm^3^Kmol^−1^ at 1.8 K.

**Figure 9:**
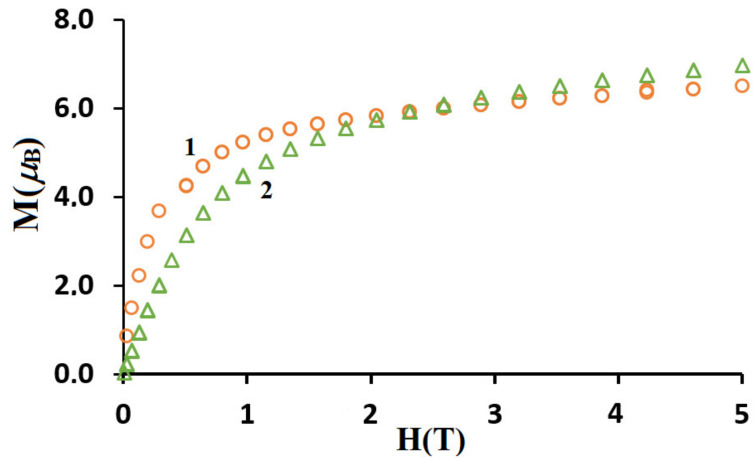
Field dependence of the magnetization for complexes **1** and **2** at 2 K.

The shape of the χ_M_*T* vs. *T* curve is strongly suggestive of the occurrence of two competitive phenomena. The decrease of χ_M_*T* on lowering of the temperature is most probably governed by the depopulation of the Ho Stark sublevels, or the presence of magnetic anisotropy, or the antiferromagnetic interaction between metal centers, while the increase of the χ_M_*T* at lower temperatures may be attributed to a ferromagnetic Cu^II^Ho^III^ coupling. For a Cu_2_^II^Er^III^ **2**, the experimental value of χ_M_*T* product at room temperature is equal to 12.18 cm^3^Kmol^−1^ and approximately corresponds to the value 12.23 cm^3^Kmol^−1^ calculated for one independent Er^III^ (^4^*I*_15/2_, *S* = 3/2, *L* = 6, *J* = 15/2, *g* = 6/5) and two independent Cu^II^ ions (*S* = 1/2, *g* = 2). As shown in Figure 9, this value decreases by lowering the temperature to 8.05 cm^3^Kmol^−1^ at 1.8 K. The reduction of χ_M_*T* at low temperature should mainly arise from the crystal field splitting of Ln^III^ ions and/or combine the contribution of the overall antiferromagnetic interactions among the metal ions. These results are compatible with the empirical investigations of heterometallic Cu^II^–4*f* compounds, in which the 4*f* ions show a spin–orbit coupling [32,61,62,63].

The *M* vs. *H* plots (at 2 K) for **1** and **2** are presented in Figure 9. The values of magnetization rise quickly at the low magnetic field whereas at the high magnetic field, the increase of magnetization is slow and linear. The magnetization reaches the values 6.5 *μ*_B_ for **1** and 7.0 *μ*_B_ for **2**, respectively, at 5T; these are far from the theoretical saturated values anticipated for one uncoupled lanthanide(III) ion and two copper(II) ions [32,61,62,63].

## 4. Conclusions

In summary, neutral, heteronuclear Cu^II^Ln^III^Cu^II^ complexes were obtained in a step-wise manner. In the crystal structures of **1** and **2**, the smaller Cu^II^ ion is exclusively coordinated to the N_2_O_2_ compartment of the hexadentate Schiff base ligand, while the O_2_O_2_ compartment accommodates a bigger Ho^III^/Er^III^ ion. The Ho^III^/Er^III^ and Cu^II^ ions are double-bridged by two phenoxo oxygen atoms of the N_2_O_4_ ligand. The complexes **1** and **2** crystallize as stable at room temperature solvates and their desolvation process is consistent with the loss of methanol molecules. The similar values of ionic radious of Ho^III^ and Er^III^ cations led to the same coordination number and the same coordination geometry of Ln^III^ ions as well as the similar spectral and thermal properties. The Cu^II^ and Ho^III^ centers are ferromagnetically coupled which is in agreement with earlier observations in similar Cu^II^Ho^III^ compounds. The structural investigations indicate that different (heterodi-, heterotri, -heterohexanuclear) coordination architectures can be received using the same Schiff base as a ligand, but changing the lanthanide(III) ions.

## Figures and Tables

**Table 1:** Details of data collection and structure refinement parameters for complexes.

Compound	1	2
CCDC	2165783	2165782
Temperature K	120(2)	298(2)
Crystal system	monoclinic	monoclinic
Space group	*P*2_1_/*c*	*P*2_1_/*c*
*a* (Å)	8.5856(3)	8.5422(5)
*b* (Å)	30.3312(12)	30.3435(15)
*c* (Å)	14.1441(6)	14.1234(7)
*β* (°)	101.114(4)	101.081(5)
Volume (Å^3^)	3614.2(2)	3592.5(3)
Z	4	4
Calculated density (g cm^−3^)	1.970	1.986
*μ* (mm^−1^)	3.419	3.573
Absorption correction	multi-scan	multi-scan
F(000)	2128	2132
Crystal size (mm)	0.22 × 0.10 × 0.05	0.20 × 0.08 × 0.05
*θ* range (°)	2.49 to 26.37	2.43 to 26.37
Reflections collected/unique	17840/7403	17336/7613
R_int_	0.0575	0.0648
Data/restraints/parameters	7403/3/545	7613/2/545
GooF on F^2^	1.032	1.015
Final R indices [I > 2σ(I)]	R1 = 0.0461, wR2 = 0.0734	R1 = 0.0516, wR2 = 0.0837
R indices (all data)	R1 = 0.0761, wR2 = 0.0833	R1 = 0.0862, wR2 = 0.0968
Largest diff. peak/hole, e Å^−3^	0.961/−0.892	2.001/−1.554

**Table 2:** The selected frequencies (cm^−1^) of absorption bands in FTIR spectra of Schiff base (**H_4_L**), Cu^II^–Ho^III^–Cu^II^ **1** and Cu^II^–Er^III^–Cu^II^ **2**.

H_4_L	1	2	Proposed Assignments
3192			*ν*(OH) + *ν*(N–H)
	2929	2924	*ν*(OH) + *ν*(CH_as_)
1632	1618	1616	*ν*(C=N)
1540, 1517	1569	1570	*ν*(C=C)
1446	1467	1465	*ν*(C=C) + *ν*(N–O)_complex_
1394	1402	1404	*ν*(C–H) + *ν*(CCC)
1355	1365	1356	*δ*(O–H)
	1285	1287	*ω*(C–H) *+δ*(O–H) + *ν*(N–O)
1233	1251	1248	*ν*(C–O)
	1218	1219	*ν*(C–O)
1189	1168	1167	*δ*(O–H)
1126	1125	1119	*ν*(C–C) + *tw*(C–H)
	1088	1087	*ν*(C–O)_methanol_
1064	1069	1069	*δ*(C–H) + *skeletal*
	1024	1024	*ν*(N–O)
896			*ρ*(C–H) + *δ*(CCC)
865	863	863	*γ*(O–H)
	782	781	*γ*(C–H) + *ν*(N–O)
711	741	734	*γ*(C–H)
	639	641	*δ*(C=C) + *ring deform.*
	614	615	*ring deform.*
	556	558	*ν*(M–O)
	538		*ν*(M–N)

**Table 3:** Selected interatomic distances and bond angles for **1** and **2**.

*Bond Lengths* (Å)			
1		2	
Cu(1)–N(1)	1.983(5)	Cu(1)–N(1)	1.977(5)
Cu(1)–N(2)	1.978(4)	Cu(1)–N(2)	1.976(5)
Cu(1)–O(1n)	2.417(4)	Cu(1)–O(1n)	2.409(6)
Cu(1)–O(2)	1.946(3)	Cu(1)–O(2)	1.957(4)
Cu(1)–O(3)	1.952(4)	Cu(1)–O(3)	1.944(4)
Cu(2)–N(3)	1.971(4)	Cu(2)–N(3)	1.959(5)
Cu(2)–N(4)	1.971(4)	Cu(2)–N(4)	1.976(5)
Cu(2)–O(6)	1.929(3)	Cu(2)–O(6)	1.913(4)
Cu(2)–O(7)	1.920(4)	Cu(2)–O(7)	1.932(4)
Cu(2)–O(4n)	2.644(4)	Cu(2)–O(4n)	2.649(5)
Ho(1)–O(1)	2.360(4)	Er(1)–O(1)	2.340(4)
Ho(1)–O(2)	2.320(4)	Er(1)–O(2)	2.303(4)
Ho(1)–O(3)	2.316(3)	Er(1)–O(3)	2.319(4)
Ho(1)–O(4)	2.355(3)	Er(1)–O(4)	2.347(4)
Ho(1)–O(5)	2.439(4)	Er(1)–O(5)	2.221(5)
Ho(1)–O(6)	2.343(4)	Er(1)–O(6)	2.304(4)
Ho(1)–O(7)	2.308(4)	Er(1)–O(7)	2.325(4)
Ho(1)–O(8)	2.242(3)	Er(1)–O(8)	2.409(5)
Ho(1)–Cu(1)	3.4977(8)	Er(1)–Cu(1)	3.4871(7)
Ho(1)–Cu(2)	3.4939(8)	Er(1)–Cu(2)	3.4831(7)
		Cu(1)–N(1)	1.978(5)
***Angles*** **(°)**			
Cu(1)–O(2)–Ho(1)	109.83(14)	Cu(1)–O(2)–Er(1)	109.65(19)
Cu(1)–O(3)–Ho(1)	109.76(15)	Cu(1)–O(3)–Er(1)	109.48(18)
O(2)–Cu(1)–O(3)	76.98(14)	O(2)–Cu(1)–O(3)	77.08(17)
Cu(2)–O(6)–Ho(1)	109.36(15)	Cu(2)–O(6)–Er(1)	111.0(2)
Cu(2)–O(7)–Ho(1)	111.13(15)	Cu(2)–O(7)–Er(1)	109.47(19)
O(6)–Ho(1)–O(7)	62.17(12)	O(6)–Er(1)–O(7)	62.28(15)
O(6)–Cu(2)–O(7)	77.20(15)	O(6)–Cu(2)–O(7)	77.04(17)
O(2)–Ho(1)–O(3)	63.12(12)	O(2)–Er(1)–O(3)	63.45(14)
*φ* ^a^	5.10	*φ* ^c^	5.23
*φ* ^b^	3.40	*φ* ^d^	3.84

^a^ The dihedral angle between the O(2)–Cu(1)–O(3) plane and the O(2)–Ho(1)–O(3) plane; ^b^ The dihedral angle between the O(6)–Cu(2)–O(7) plane and the O(6)–Ho(1)–O(7) plane; ^c^ The dihedral angle between the O(2)–Cu(1)–O(3) plane and the O(2)–Er(1)–O(3) plane; ^d^ The dihedral angle between the O(6)–Cu(2)–O(7) plane and the O(6)–Er(1)–O(7) plane.

**Table 4:** The values of a long Cu-O_nitrate_ bond length [50].

Refcode	Cu-O_nitrate_ Bond Length [Å]	Reference
AZIROS	2.626(4)	[28]
AZIRIM	2.646(4)	[28]
CERZAD	2.785(4)	[40]
CAWYEG	2.833(7)	[51]
DIFTET	2.759(4)	[52]
FUBTEE	2.714(5)	[53]
HUYYOS	2.741(3)	[54]
KOCMOE	2.643(2)	[55]
MESBAQ	2.718(1)	[56]
MESBEU	2.734(6)	[56]
MIWLEL	2.762(4)	[57]
SAJTAB	2.701(2)	[58]

## Data Availability

The data presented in this study are available on request from the corresponding author. The CIF files have been deposited in the Cambridge Crystallographic Data Center (CCDC). These data can be obtained free of charge from The Cambridge Crystallographic Data Centre via www.ccdc.cam.ac.uk/data_request/cif (or from the CCDC, 12 Union Road, Cambridge CB2 1EZ, UK; Fax: +44-1223-336033; E-mail: deposit@ccdc.cam.ac.uk).

## Supplementary Materials

The following supporting information can be downloaded at: https://www.mdpi.com/article/10.3390/ma15124299/s1, Figure S1: FTIR spectra of the free Schiff base H_4_L and complexes **1** and **2**, Figure S2: The molecular structure of **2**. Displacement ellipsoids are drawn at the 30% probability level, Figure S3: Coordination polyhedra of Cu(II) and Er(III) cations in the trinuclear complex **2**, Figure S4: (a) A partial viewed along the *b*-axis direction of the crystal packing of **2** with hydrogen bonds shown as dashed lines; (b) A partial viewed along the *a*-axis direction of the crystal packing of **2** with hydrogen bonds shown as dashed lines, Figure S5: The overall crystal packing of compound **1** showing formation of 3D supramolecular structure, viewed along the *a*-axis. Hanging contacts were omitted for clarity, Figure S6: The overall crystal packing of compound **2** showing formation of 3D supramolecular structure, viewed along the *c*-axis. Hanging contacts were omitted for clarity, Figure S7: TG and DCS curves of thermal decomposition of the complex **1** in air, Figure S8: FTIR spectra of gaseous products involved during the complex **2** decomposition, Figure S9: The X-ray powder diffraction patterns of the final products of complex **1** decomposition in air, Figure S10: The X-ray powder diffraction patterns of the final products of complex **2** decomposition in air, Table S1: Hydrogen-bond geometry [Å, °] for compounds **1** and **2**.
