# Supplementary material for: New Heterotrinuclear CuIILnIIICuII (Ln = Ho, Er) Compounds with the Schiff Base: Syntheses, Structural Characterization, Thermal and Magnetic Properties
Source: Materials (Basel). 2022 Jun 17;15(12):4299. doi: 10.3390/ma15124299 (PMC9231215; doi:10.3390/ma15124299)
Supplement: Supplementary file 1 [file materials-15-04299-s001.zip › materials-1703837-supplementary.pdf]

New heterotrinuclear $Cu^{II}Ln^{III}Cu^{II}$ (Ln = Ho, Er) compounds with Schiff base: syntheses, structural characterization, thermal and magnetic properties.

Beata Cristóvão*, Dariusz Osypiuk, Agata Bartyzel

Department of General and Coordination Chemistry and Crystallography, Institute of Chemical Sciences, Faculty of Chemistry, Maria Curie-Sklodowska University in Lublin, Maria Curie-Sklodowska sq. 2, 20-031 Lublin, Poland, dariusz.osypiuk@mail.umcs.pl (D.O); agata.bartyzel@mail.umcs.pl (A.B.)
*Correspondence: beata.cristovao@mail.umcs.pl (B.C.)

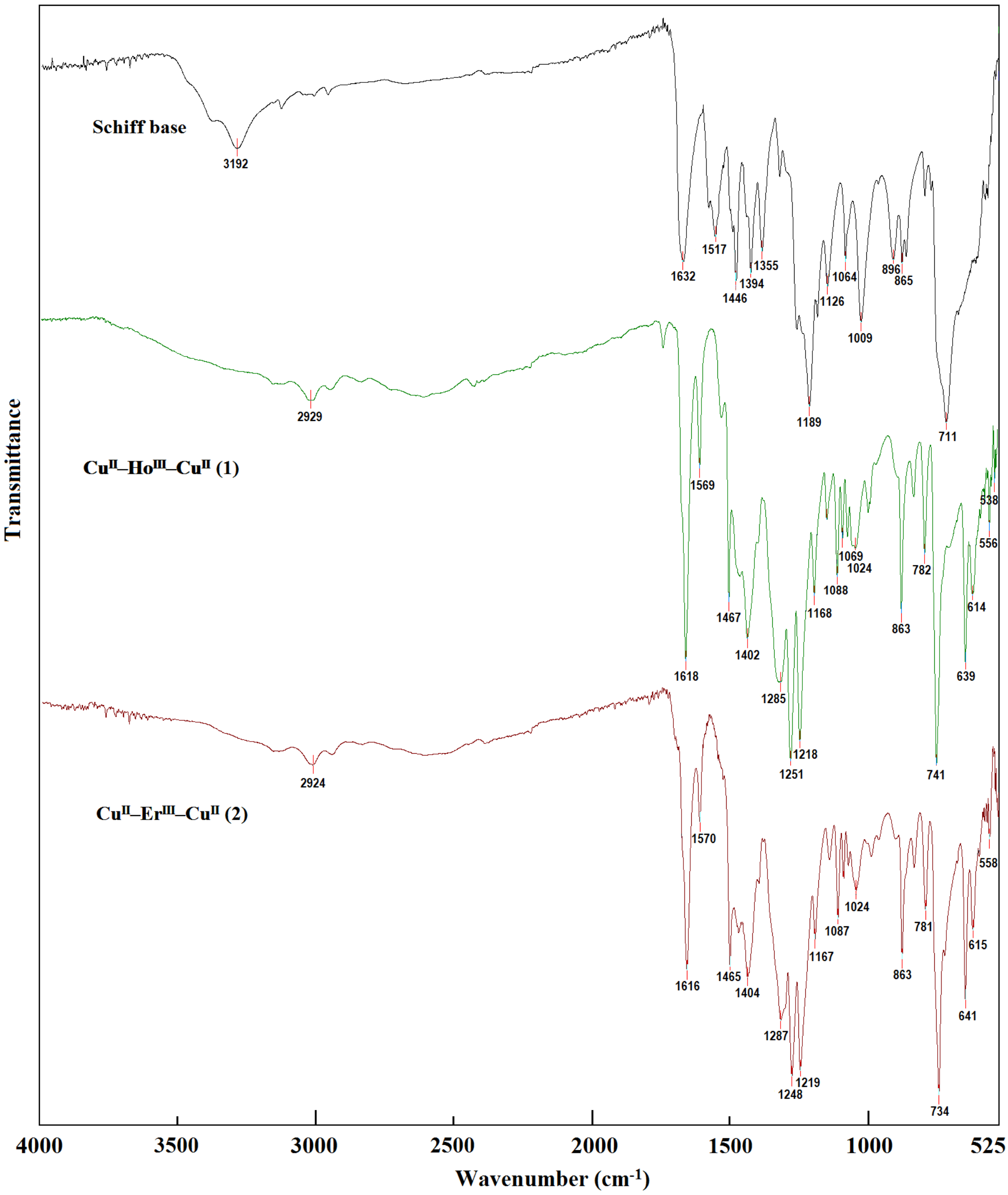

Figure S1. FTIR spectra of the free Schiff base $\mathbf{H_4L^1}$ and complexes **1** and **2**.

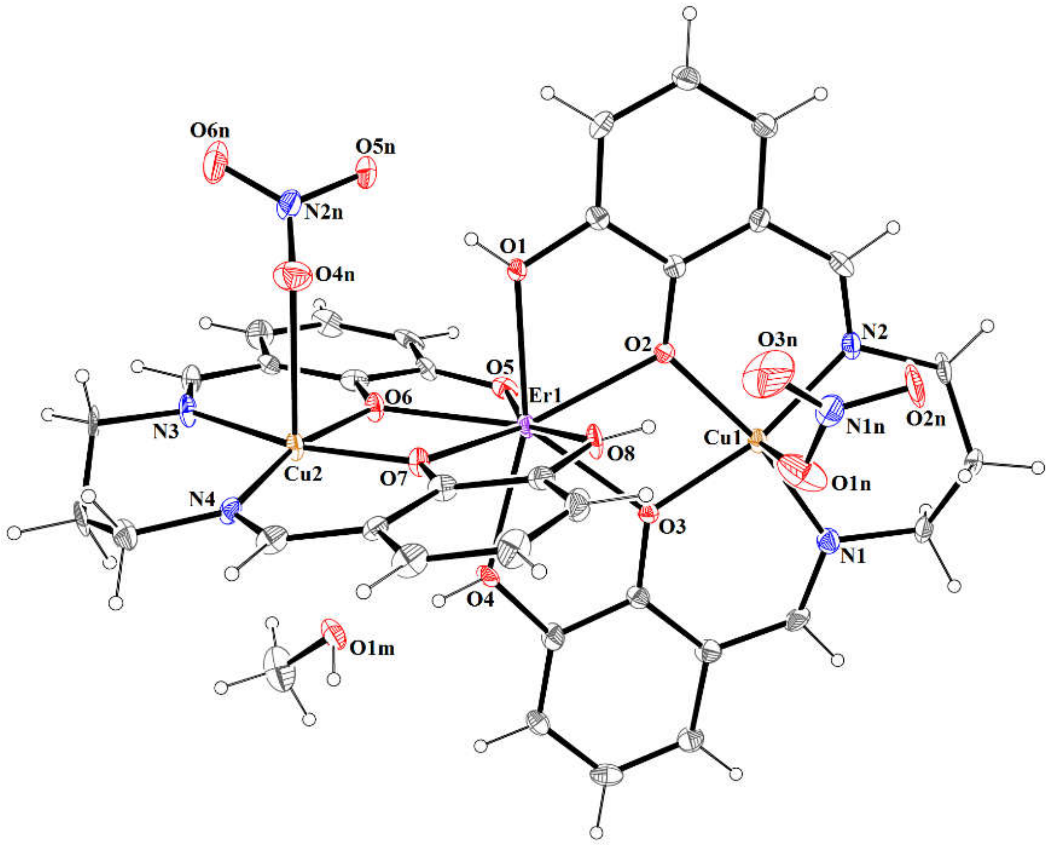

Figure S2. The molecular structure of **2**. Displacement ellipsoids are drawn at the 30% probability level.

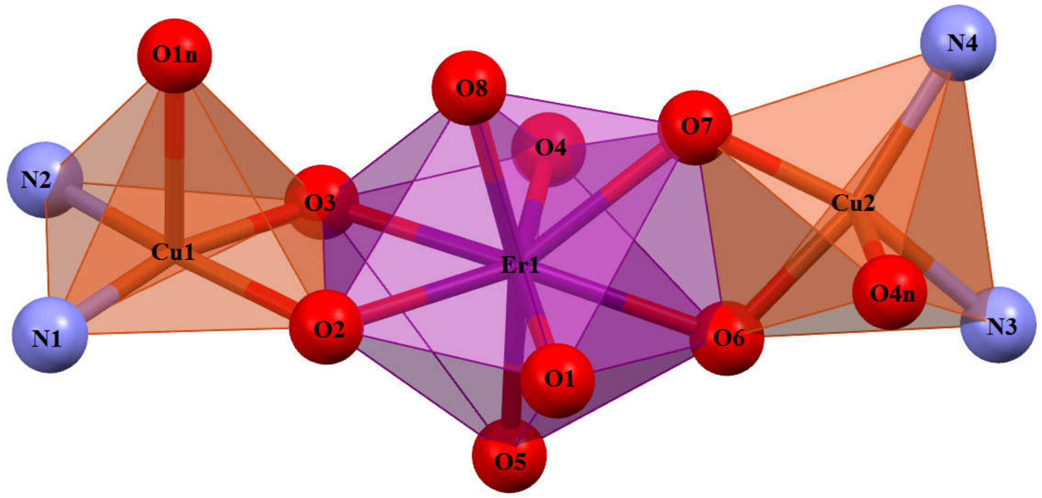

Figure S3. Coordination polyhedra of Cu(II) and Er(III) cations in the trinuclear complex **2**.

Table S1. Hydrogen-bond geometry [Å, °] for compounds **1** and **2**.

D-H···A	d(D-H)	d(H···A)	d(D···A)	∠ DHA
**1**				
O(4)–H(4o)···O(5n)	0.81	1.79	2.603(6)	172
O(1)–H(1o)···O(1m)	0.82(2)	1.74(3)	2.533(6)	163(5)
O(1m)–H(1m)···O(5n)[1a]	0.81(2)	1.88(2)	2.676(6)	169(6)
O(5)–H(5o)···O(1n)	0.82(2)	2.29(4)	3.042(6)	152(7)
O(5)–H(5o)···O(2n)	0.82(2)	1.96(4)	2.684(7)	148(7)
C(11)–H(11)···O(3n)[1b]	0.95	2.34	3.237(7)	158
C(25)–H(25A)···O(6n)[1a]	0.99	2.49	3.336(7)	144
C(28)–H(28)···O(8)[1c]	0.95	2.33	3.247(6)	163
**2**				
O(4)-H(4o)···O(1m)	0.82	1.71	2.520(5)	165
O(1)-H(1o)···O(5n)	0.81(2)	1.80(3)	2.594(6)	167(8)
O(1m)-H(1M)···O(5n)[2a]	0.83(7)	1.85(8)	2.668(7)	169(8)
O(8)-H(8o)···O(1n)	0.81(2)	2.39(5)	2.998(7)	133(6)
O(8)-H(8o)···O(3n)	0.81(2)	1.94(2)	2.748(8)	170(7)
C(7)-H(7)···O(2n)[2b]	0.93	2.36	3.234(8)	156
C(24)-H(24)···O(5)[2c]	0.93	2.37	3.256(8)	160
C(27)-H(27A)···O(6n)[2a]	0.97	2.49	3.322(9)	144
C16--H16···O5[2a]	0.93	2.49	3.205(8)	134

Symmetry codes: compound **1** - (1a) x-1,y,z; (1b) -x+2,-y+1,-z+2; (1c) x,-y+3/2,z-1/2;
compound **2** - (2a) x-1,y,z; (2b) -x+2,-y+1,-z+2; (2c) x,-y+1/2,z-1/2

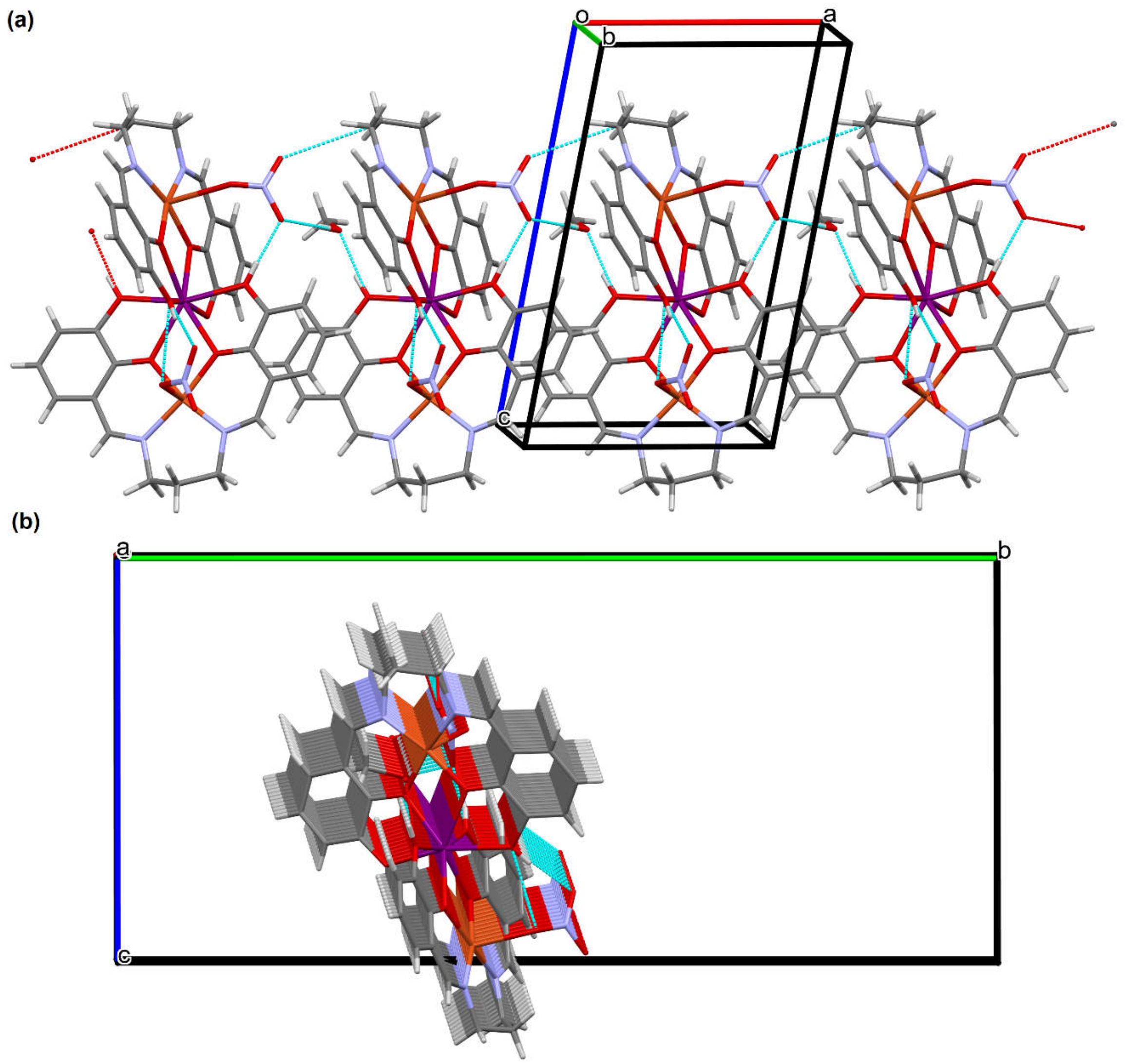

Figure S4. (**a)** A partial viewed along the *b*-axis direction of the crystal packing of **2** with hydrogen bonds shown as dashed lines. (**b)** A partial viewed along the *a*-axis direction of the crystal packing of **2** with hydrogen bonds shown as dashed lines.

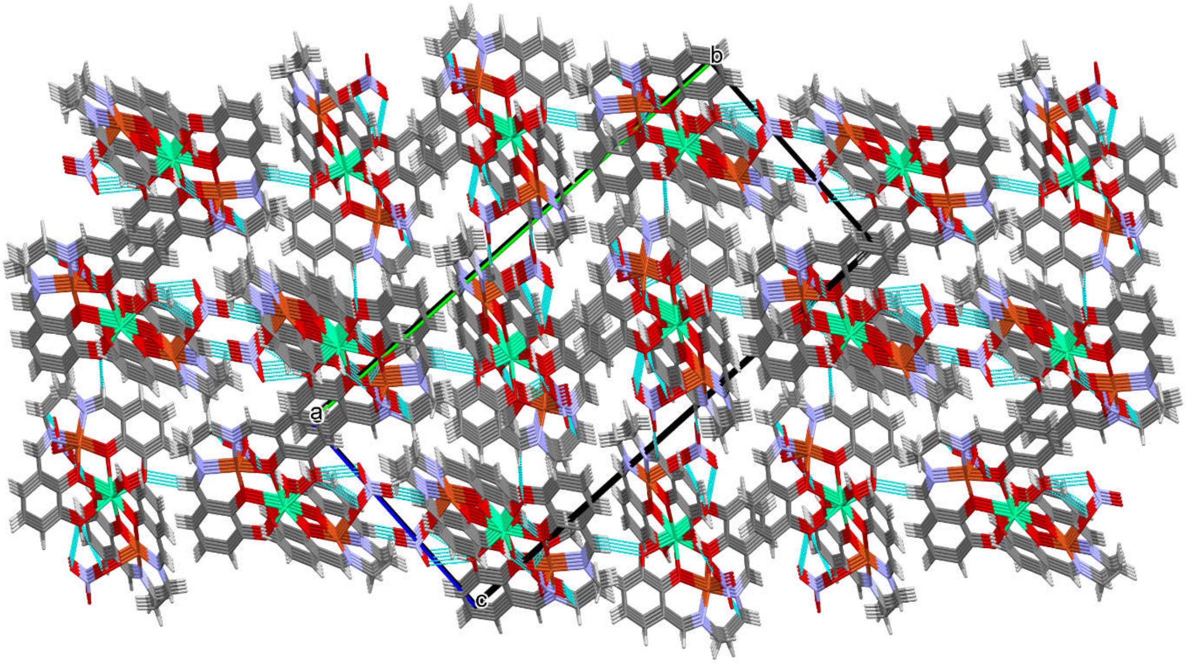

Figure S5. The overall crystal packing of compound **1** showing formation of 3D supramolecular structure, viewed along the *a*-axis. Hanging contacts were omitted for clarity.

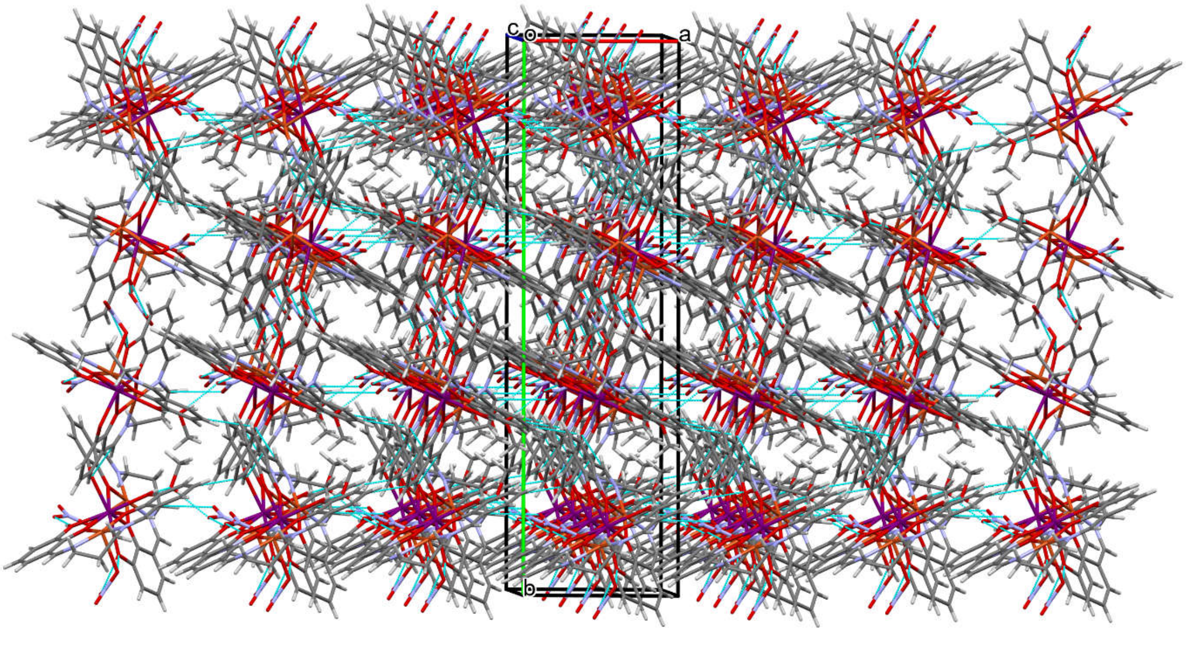

Figure S6. The overall crystal packing of compound **2** showing formation of 3D supramolecular structure, viewed along the *c*-axis. Hanging contacts were omitted for clarity.

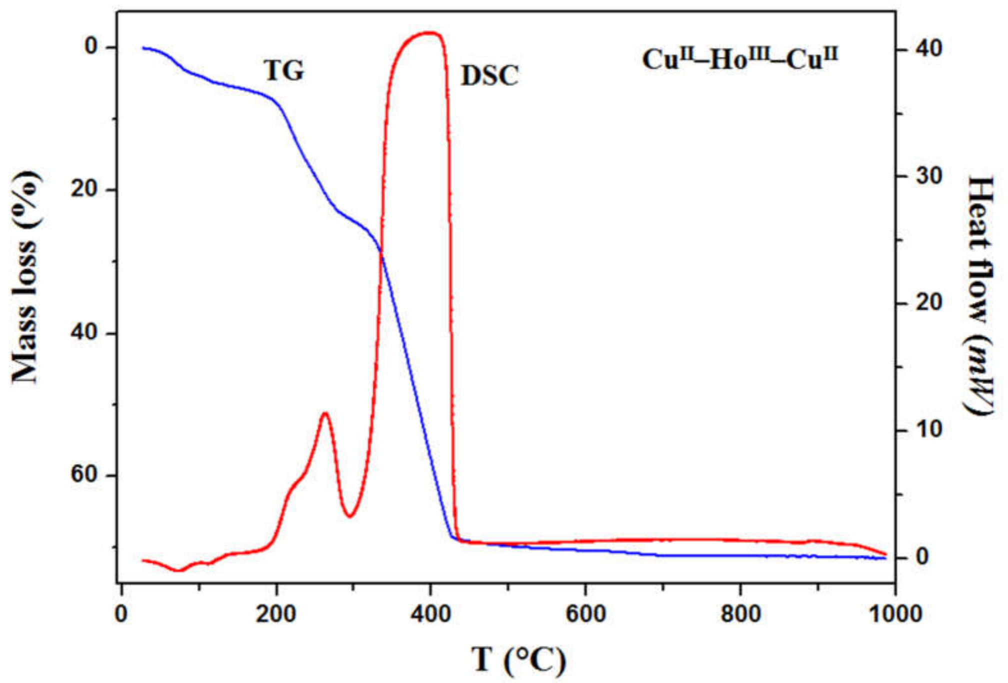

Figure S7. TG and DCS curves of thermal decomposition of the complex **1** in air.

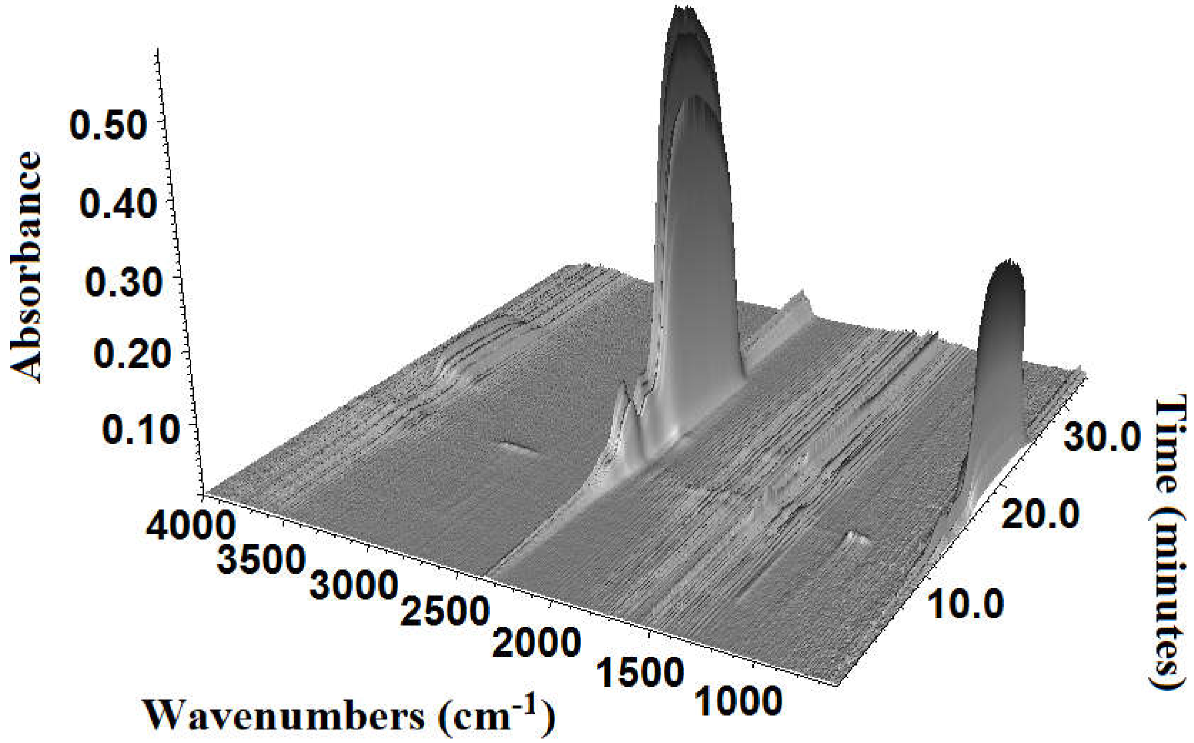

Figure S8. FTIR spectra of gaseous products involved during of the complex **2** decomposition.

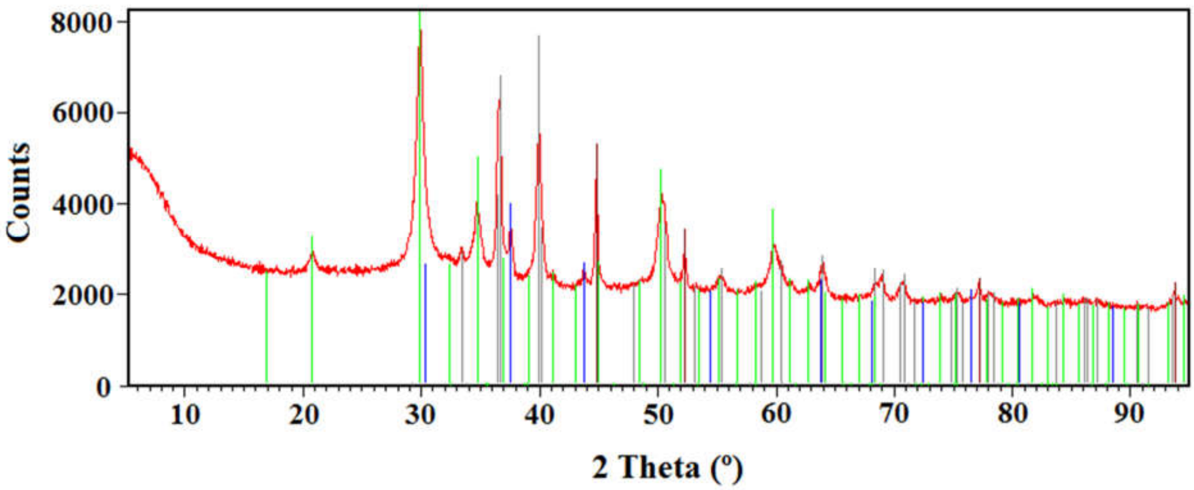

Figure S9. The X-ray powder diffraction patterns of the final products of complex **1** decomposition in air.

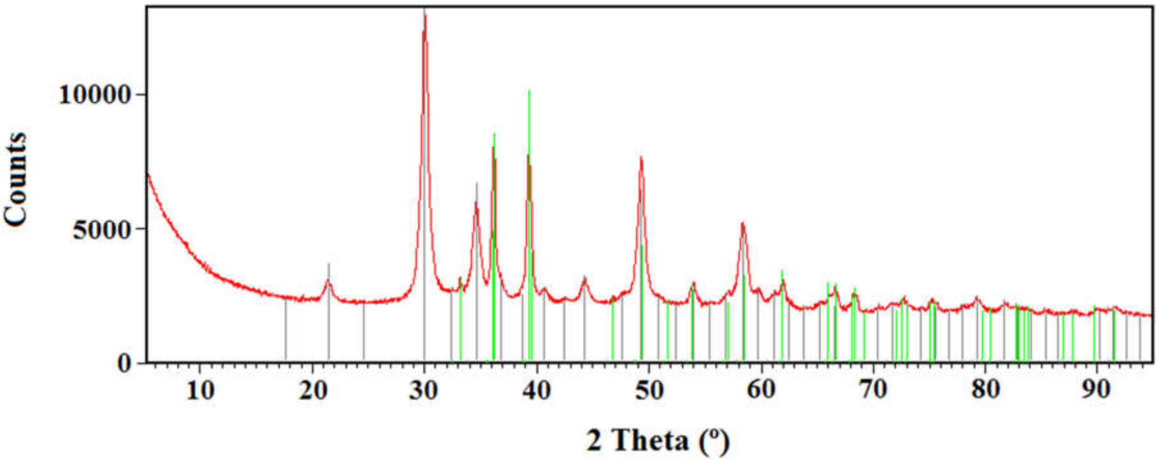

Figure S10. The X-ray powder diffraction patterns of the final products of complex **2** decomposition in air.
